# Reflections on the gamma delta T cell conference in Toronto 2025 – human therapies, mouse models and white elephants in the room

**DOI:** 10.17179/excli2025-8769

**Published:** 2025-10-21

**Authors:** Leander Sollberg, Charlotte Esser

**Affiliations:** 1 IUF - Leibniz Research Institute for Environmental Medicine, Duesseldorf, Germany

## ⁯⁯⁯

Immunologists are well-known, sometimes notoriously so, for identifying ever more refined subpopulations of immune cells. It all started with the observation of leukocytes versus erythrocytes in blood in the 19^th^ century, e.g., by Rudolf Virchow, but later moved on to the identification of B and T cells, macrophages, dendritic cells, innate lymphoid cells, and many more. These cells have been further characterized into functional sub-sub-subsets, mostly via their surface markers. They are known as “clusters of differentiation” (CD), molecules that are identifiable by antibodies, and which are simply numbered once discovered. By now, more than 371 such CD-molecules are recognized and used. Other markers include cytokine or chemokine receptors, among others. Moreover, in the T cell field, two major lineages exist due to the genetic use of either the alpha and beta or gamma and delta receptor chain loci (located on different chromosomes) for generating the unique rearranged T cell receptor of each cell. Unsurprisingly, these αß or γδ T cells also differ markedly in function, tissue-specificity, and developmental and evolutionary aspects. While it is possible to say that most of the literature on T cells deals with the conventional αß T cells, the γδ T cells have their aficionados and a stable niche in the literature. Interest in them started with their discovery and description by immunology doyens like Adrian Hayday, Joseph Heilig/Susumu Tonegawa, Philippa Marrack/John Kappler, or James Allison in the 1980s (Hayday et al., 1985[4]). This drew considerable interest in γδ T cells, and subsequent research identified their roles as rapid responders, capable of secreting cytokines, their ability to recognize antigens independently of MHC, and their function in protecting skin or gut epithelia (Mensurado et al., 2023[6]; Ribot et al., 2021[9]). Following a period of diminished interest, research attention increased once the mechanisms by which γδ T cells recognize antigens became better understood and evidence emerged regarding their unique therapeutic potential against certain cancers (Girardi et al., 2020[2]). At the same time, it became clear that subsets of γδ T cells are resident in barrier tissues and have unique features and an invariant T cell receptor. A small conference on γδ T cells was organized already in 2004 by Rebecca O´Brien and Willi Born in Denver, Colorado, USA, and immediately became the biennial go-to event around the globe for γδ T cell researchers. The most recent event took place in Toronto, Canada, in May 2025 and attracted over 300 participants who were primarily interested in mouse and human γδ T cells with a major focus on γδ T cell-based cancer therapies. In her opening keynote, Erin Adams challenged the dogma that γδ T cells do not recognize MHC molecules and emphasized that such claims should be taken with caution. Of particular interest to us was the presentation by Nicolas Veland, who examined the relationship between the γδ T cell receptor, cell metabolism and effects on gene expression and thus the killing capacity of γδ T cells. Notably, a significant number of younger scientists attended the conference for the first time, highlighting the field´s increasing appeal to the next generation of immunologists. One of the authors of this paper, Leander Sollberg, an early career researcher working on murine skin-specific resident γδ T cells, attended and can vouch for the rich discussions and enthusiastic atmosphere of this small community of basic and clinical immunologists.

He returned, however, intrigued once more how distinct mouse γδ T cells (especially skin-resident) are from human γδ T cells. This, of course, is an obstacle for those mainly interested in the biomedical translational trajectory. They need to justify themselves when they use mice as a model. Of course, for us and many other immunologists, murine γδ T cells can be a research object worthy of their own (Merches et al., 2020[7]; Häselbarth et al., 2020[3]). In addition, there is the intriguing evolution of γδ T cells, which in our opinion deserves a lot more attention if one wants to understand γδ T cell biology and function. Yet, the evolution of γδ T cells and species-specific usage, frequency, and functions are a white elephant in the current research room.

For instance, in mouse epidermis, all γδ T cells use a particular Vγ5 chain to sense stress, kill cancer cells, or prevent overshooting inflammation. For this, they keep in contact to the surrounding keratinocytes with many long dendrites. Guided by the molecule Skint1, they migrate into the skin after being formed during a narrow developmental window in the fetal thymus, then expand locally and keep up their numbers by slow division, although with age they decrease, nonetheless. In humans, dermal γδ T cells with a Vδ1 receptor are thought to be DETC´s functional analog for immunosurveillance (Toulon et al., 2009[10]). However, several notable differences exist: these cells lack dendrites, circulate between the blood and skin, and in some cases, another subset of γδ T cells, Vγ9Vδ2 cells, may immigrate into the skin and play an equally significant role in epithelial defense against infections and inflammation. What about other species then? There is much less known as evident from Table 1(Tab. 1). Old World monkeys have also dendritic γδ T cells in the epidermis, but with a variant TCR and thus also differ from murine DETCs (Mohamed et al., 2015[8]). Somewhat overlooked in mainstream immunology is the fact that γδ T cells are the dominant T cell lineage in ruminants, which was already reported in the 1980s. Bovine γδ T cells are abundant and diverse, have unique co-receptors (notably the ruminant-specific multi-gene WC1 family), and feature pathogen recognition and regulatory functions beyond those observed in humans or mice (Crocker et al., 1993[1]). However, cows are big animals, and genetic tools, knock-out animal models, and reagents are often missing or limited, hindering research. There are more genes for the γδ receptor in ruminants, and thus they differ sharply from the more restricted repertoires of humans and mice. Other artiodactyls, a taxonomic order including pigs, sheep, goats, giraffes, and deer, have variations in certain γδ TCR segments, which highlight divergent evolutionary solutions for phosphoantigen recognition. All in all, the regulatory and effector roles of γδ T cells are fundamental, but the molecular mechanisms, especially relating to co-receptors and ligand specificity, can differ dramatically between species (Holderness et al., 2013[5]).

For early career scientists, having a broad understanding of their research field is vital, as it informs good choices. An important choice for immunologists and γδ T cell researchers is, of course, whether to pursue basic or translational/applied research. Should one move into areas of γδ T cell research, where translation and therapy are visibly within reach and where pharmaceutical players are becoming active? Or go the route down to basic research and take a broader view of the biology and evolution of γδ T cells, irrespective of any eventual biomedical applicability?

To aid this decision, we think it can be helpful to highlight the knowledge gap regarding the significance of γδ T cells in other species, as discussed above. Moreover, we posit that the integration of research findings from different species is needed to create a more unified picture and identify models that are evolutionarily closer to humans (or confirm the mouse as a suitable model). What is the reason that a mechanism such as the immune system developed so differently in vertebrates? Do γδ T cell-rich species face unique environmental pressure that shapes their immune architecture? Many questions remain unresolved, yet attempting to answer them could be valuable.

Comparative immunology reminds us that evolutionary differences are not a footnote but a crucial factor in the architecture of the immune system. The selection of a model organism is therefore not just about technical convenience, but a conceptual decision that shapes the relevance and impact of our findings. Precisely because the immune system is such a complex and finely tuned system, it may make sense to look beyond traditional model organisms, even for applied sciences. Only in this way can we truly sharpen our questions and recognize the evolutionary subtleties that often vary surprisingly between species, despite a common basis.

## Acknowledgement

We gratefully acknowledge support of our own γδ T cell research by the Deutsche Forschungsgemeinschaft, grant ES103/10-1. The IUF is funded by the federal and state governments - the Ministry of Culture and Science of North Rhine-Westphalia (MKW) and the Federal Ministry of Research, Technology and Space (BMFTR).

## Figures and Tables

**Table 1:**
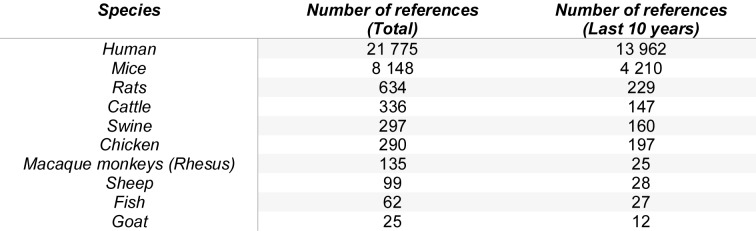
Number of γδ T cell-related publications by species. Search terms used: gamma delta T cell AND [species][MeSH], queried via PubMed in July 18, 2025.
